# Supplementary material for: Analytic comparison between three high-throughput commercial SARS-CoV-2 antibody assays reveals minor discrepancies in a high-incidence population
Source: Sci Rep. 2021 Jun 4;11:11837. doi: 10.1038/s41598-021-91235-x (PMC8178338; doi:10.1038/s41598-021-91235-x)
Supplement: Supplementary file 1 — Supplementary Information. [file 41598_2021_91235_MOESM1_ESM.docx]

**Supplementary Information**

**Analytic comparison between three high-throughput commercial SARS-CoV-2 antibody assays reveals minor discrepancies in a high-incidence population**

Gheyath K. Nasrallah*, Soha R. Dargham, Farah Shurrab, Duaa W. Al-Sadeq, Hadeel Al-Jighefee, Hiam Chemaitelly, Zaina Al Kanaani, Abdullatif Al Khal, Einas Al Kuwari, Peter Coyle, Andrew Jeremijenko, Anvar Hassan Kaleeckal, Ali Nizar Latif, Riyazuddin Mohammad Shaik, Hanan F. Abdul Rahim, Hadi M. Yassine, Mohamed G. Al Kuwari, Hamda Qotba, Hamad Eid Al Romaihi, Patrick Tang, Roberto Bertollini, Mohamed H. Al-Thani, Asmaa A. Althani and Laith J. Abu-Raddad*

*Correspondence:

Gheyath K. Nasrallah, Department of Biomedical Science, College of Health Sciences, Qatar University, Doha, Qatar. Women’s Science building, C01, Tel: +974 4403 4817, Fax: +974-4403-1351, P.O Box: 2713, email: [gheyath.nasrallah@qu.edu.qa](mailto:gheyath.nasrallah@qu.edu.qa).

Professor Laith J. Abu-Raddad, Infectious Disease Epidemiology Group, Weill Cornell Medicine - Qatar, Qatar Foundation - Education City, P.O. Box 24144, Doha, Qatar. Telephone: +(974) 4492-8321. Fax: +(974) 4492-8333. E-mail: [lja2002@qatar-med.cornell.edu](mailto:lja2002@qatar-med.cornell.edu).

**Supplementary Table S1.** Characteristics of all specimens tested using the Mindray CL-900i anti-SARS-CoV-2 IgG, BioMérieux VidasIII anti-SARS-CoV-2 IgG, and Roche Elecsys Anti SARS-CoV-2 antibody testing.

| **Specimen number^%^** | **Record of PCR positive test prior to study** | | | **Date of blood and naso-pharyngeal swab specimens’ collection at time of study** | **PCR test at time of study** | | **Mindray CL-900i  anti-SARS-CoV-2 IgG^*^** | | **BioMérieux VidasIII anti-SARS-CoV-2 IgG^**^** | | **Roche Elecsys Anti SARS-CoV-2^***^** | | **Symptomatic at time of study^#^** | **Severity at or right after time of study^$^** |
| --- | --- | --- | --- | --- | --- | --- | --- | --- | --- | --- | --- | --- | --- | --- |
|  | **Test result** | **Ct value** | **Date of first positive swab** |  | **Test result** | **Ct value** | **Optical density** | **Test Result** | **Optical density** | **Test Result** | **Optical density** | **Test Result** |  |  |
| 1 | Positive | 31.05 | 14-May-20 | 10-Aug-20 | Negative |  | 517.46 | Positive | 32.66 | Positive | 41.03 | Positive | No | N/A |
| 2 | Positive | 25.22 | 17-May-20 | 23-Jul-20 | Negative |  | 574.15 | Positive | 47.50 | Positive | 68.50 | Positive | No | N/A |
| 3 | Positive | 14.91 | 9-Apr-20 | 27-Jul-20 | Negative |  | 64.43 | Positive | 10.06 | Positive | 105.70 | Positive | No | N/A |
| 4 | Positive | 20.40 | 21-Jun-20 | 27-Jul-20 | Negative |  | 630.73 | Positive | 38.82 | Positive | 15.14 | Positive | No | N/A |
| 5 | Positive | 14.82 | 27-May-20 | 29-Jul-20 | Negative |  | 106.30 | Positive | 10.75 | Positive | 19.38 | Positive | No | N/A |
| 6 | Positive | 34.72 | 25-Jul-20 | 23-Aug-20 | Negative |  | 10.01 | Positive | 2.00 | Positive | 16.50 | Positive | No | N/A |
| 7 | Positive | 25.56 | 24-Jun-20 | 29-Jul-20 | Negative |  | 216.63 | Positive | 48.01 | Positive | 20.61 | Positive | Not reported | N/A |
| 8 | Positive | 18.99 | 21-May-20 | 27-Jul-20 | Negative |  | 366.48 | Positive | 14.45 | Positive | 101.30 | Positive | No | N/A |
| 9 | Positive | 36.82 | 25-Jul-20 | 27-Jul-20 | Negative |  | 16.11 | Positive | 1.13 | Positive | 26.70 | Positive | No | N/A |
| 10 | Positive | 31.49 | 20-Apr-20 | 27-Jul-20 | Negative |  | 116.04 | Positive | 8.90 | Positive | 98.78 | Positive | No | N/A |
| 11 | Positive | 35.27 | 15-Jul-20 | 23-Jul-20 | Negative |  | 18.65 | Positive | 4.09 | Positive | 7.68 | Positive | No | N/A |
| 12 | Positive | 19.22 | 20-Apr-20 | 26-Jul-20 | Negative |  | 42.91 | Positive | 2.09 | Positive | 15.79 | Positive | No | N/A |
| 13 | Positive | 25.80 | 7-May-20 | 10-Aug-20 | Negative |  | 13.37 | Positive | 2.40 | Positive | 37.70 | Positive | No | N/A |
| 14 | Positive | 17.49 | 4-May-20 | 10-Aug-20 | Negative |  | 23.77 | Positive | 1.78 | Positive | 31.71 | Positive | No | N/A |
| 15 | Positive | 15.81 | 19-May-20 | 29-Jul-20 | Negative |  | 119.78 | Positive | 4.04 | Positive | 62.33 | Positive | No | N/A |
| 16 | Positive | 34.94 | 9-Jun-20 | 29-Jul-20 | Negative |  | 26.63 | Positive | 2.69 | Positive | 34.38 | Positive | No | N/A |
| 17 | Positive | 15.96 | 23-Jul-20 | 27-Jul-20 | Declined testing |  | 0.27 | Negative | 0.08 | Negative | 0.07 | Negative | No | N/A |
| 18 | N/A |  |  | 27-Jul-20 | Negative |  | 0.27 | Negative | 0.05 | Negative | 0.07 | Negative | No | N/A |
| 19 | N/A |  |  | 29-Jul-20 | Negative |  | 28.65 | Positive | 2.70 | Positive | 9.21 | Positive | Not reported | N/A |
| 20 | N/A |  |  | 10-Aug-20 | Negative |  | 3.76 | Negative | 5.86 | Positive | 0.48 | Negative | No | N/A |
| 21 | N/A |  |  | 10-Aug-20 | Negative |  | 111.72 | Positive | 0.45 | Negative | 67.27 | Positive | No | N/A |
| 22 | N/A |  |  | 6-Aug-20 | Positive | 21.62 | 3.74 | Negative | 0.10 | Negative | 8.40 | Positive | Symptomatic | Severe |
| 23 | N/A |  |  | 10-Aug-20 | Positive | 23.33 | 0.35 | Negative | 0.08 | Negative | 0.08 | Negative | No | N/A |
| 24 | N/A |  |  | 23-Jul-20 | Negative |  | 0.17 | Negative | 0.38 | Negative | 0.07 | Negative | No | N/A |
| 25 | N/A |  |  | 23-Jul-20 | Negative |  | 1.40 | Negative | 0.00 | Negative | 0.07 | Negative | No | N/A |
| 26 | N/A |  |  | 10-Aug-20 | Negative |  | 84.85 | Positive | 6.22 | Positive | 124.00 | Positive | No | N/A |
| 27 | N/A |  |  | 27-Jul-20 | Negative |  | 0.68 | Negative | 0.00 | Negative | 0.07 | Negative | No | N/A |
| 28 | N/A |  |  | 11-Aug-20 | Negative |  | 30.09 | Positive | 4.42 | Positive | 121.00 | Positive | No | N/A |
| 29 | N/A |  |  | 27-Jul-20 | Negative |  | 5.81 | Negative | 1.08 | Positive | 5.62 | Positive | No | N/A |
| 30 | N/A |  |  | 11-Aug-20 | Negative |  | 18.64 | Positive | 6.26 | Positive | 11.24 | Positive | No | N/A |
| 31 | N/A |  |  | 27-Jul-20 | Negative |  | 0.42 | Negative | 0.13 | Negative | 0.07 | Negative | No | N/A |
| 32 | N/A |  |  | 23-Jul-20 | Negative |  | 0.32 | Negative | 0.00 | Negative | 0.07 | Negative | No | N/A |
| 33 | N/A |  |  | 29-Jul-20 | Negative |  | 212.6 | Positive | 27.55 | Positive | 76.36 | Positive | No | N/A |
| 34 | N/A |  |  | 29-Jul-20 | Negative |  | 78.55 | Positive | 3.71 | Positive | 135.90 | Positive | No | N/A |
| 35 | N/A |  |  | 10-Aug-20 | Negative |  | 86.06 | Positive | 7.75 | Positive | 61.33 | Positive | No | N/A |
| 36 | N/A |  |  | 28-Jul-20 | Negative |  | 127.23 | Positive | 14.93 | Positive | 75.75 | Positive | No | N/A |
| 37 | N/A |  |  | 10-Aug-20 | Negative |  | 7.77 | Negative | 0.47 | Negative | 3.66 | Positive | No | N/A |
| 38 | N/A |  |  | 1-Sep-20 | Negative |  | 173.35 | Positive | 4.98 | Positive | 138 | Positive | Not reported | N/A |
| 39 | N/A |  |  | 10-Aug-20 | Negative |  | 1.88 | Negative | 0.05 | Negative | 0.08 | Negative | No | N/A |
| 40 | N/A |  |  | 29-Jul-20 | Negative |  | 20.24 | Positive | 0.01 | Negative | 0.16 | Negative | No | N/A |
| 41 | N/A |  |  | 27-Jul-20 | Negative |  | 125.97 | Positive | 11.06 | Positive | 133.90 | Positive | No | N/A |
| 42 | N/A |  |  | 23-Jul-20 | Negative |  | 388.67 | Positive | 18.01 | Positive | 135.20 | Positive | No | N/A |
| 43 | N/A |  |  | 27-Jul-20 | Negative |  | 0.13 | Negative | 0.04 | Negative | 0.07 | Negative | No | N/A |
| 44 | N/A |  |  | 26-Jul-20 | Negative |  | 34.23 | Positive | 7.74 | Positive | 60.48 | Positive | Not reported | N/A |
| 45 | N/A |  |  | 28-Jul-20 | Negative |  | 80.19 | Positive | 7.48 | Positive | 30.35 | Positive | No | N/A |
| 46 | N/A |  |  | 27-Jul-20 | Positive | 31.48 | 46.64 | Positive | 2.71 | Positive | 21.66 | Positive | No | N/A |
| 47 | N/A |  |  | 27-Jul-20 | Negative |  | 0.28 | Negative | 0.00 | Negative | 0.07 | Negative | No | N/A |
| 48 | N/A |  |  | 29-Jul-20 | Negative |  | 0.58 | Negative | 0.09 | Negative | 0.07 | Negative | No | N/A |
| 49 | N/A |  |  | 28-Jul-20 | Negative |  | 28.40 | Positive | 3.60 | Positive | 76.52 | Positive | No | N/A |
| 50 | N/A |  |  | 11-Aug-20 | Negative |  | 31.25 | Positive | 0.04 | Negative | 0.09 | Negative | No | N/A |
| 51 | N/A |  |  | 28-Jul-20 | Negative |  | 0.14 | Negative | 0.07 | Negative | 0.07 | Negative | No | N/A |
| 52 | N/A |  |  | 10-Aug-20 | Positive | 19.01 | 0.46 | Negative | 0.00 | Negative | 0.09 | Negative | No | N/A |
| 53 | N/A |  |  | 26-Jul-20 | Negative |  | 0.23 | Negative | 0.02 | Negative | 0.07 | Negative | Not reported | N/A |
| 54 | N/A |  |  | 29-Jul-20 | Negative |  | 0.47 | Negative | 0.08 | Negative | 0.07 | Negative | No | N/A |
| 55 | N/A |  |  | 27-Jul-20 | Negative |  | 238.12 | Positive | 38.23 | Positive | 98.81 | Positive | No | N/A |
| 56 | N/A |  |  | 27-Jul-20 | Negative |  | 6.58 | Negative | 0.00 | Negative | 0.08 | Negative | No | N/A |
| 57 | N/A |  |  | 10-Aug-20 | Negative |  | 80.86 | Positive | 15.63 | Positive | 83.95 | Positive | No | N/A |
| 58 | N/A |  |  | 10-Aug-20 | Negative |  | 2.04 | Negative | 0.03 | Negative | 0.08 | Negative | No | N/A |
| 59 | N/A |  |  | 29-Jul-20 | Negative |  | 519.89 | Positive | 8.51 | Positive | 37.98 | Positive | No | N/A |
| 60 | N/A |  |  | 10-Aug-20 | Positive | 29.41 | 2.01 | Negative | 0.06 | Negative | 0.42 | Negative | No | N/A |
| 61 | N/A |  |  | 29-Jul-20 | Negative |  | 135.05 | Positive | 0.45 | Negative | 38.01 | Positive | No | N/A |
| 62 | N/A |  |  | 10-Aug-20 | Positive | 21.21 | 0.00 | Negative | 0.09 | Negative | 0.08 | Negative | No | N/A |
| 63 | N/A |  |  | 27-Jul-20 | Negative |  | 0.00 | Negative | 0.05 | Negative | 0.07 | Negative | No | N/A |
| 64 | N/A |  |  | 29-Jul-20 | Negative |  | 25.92 | Positive | 5.73 | Positive | 6.17 | Positive | Not reported | N/A |
| 65 | N/A |  |  | 29-Jul-20 | Positive | 18.9 | 0.63 | Negative | 0.00 | Negative | 0.07 | Negative | No | N/A |
| 66 | N/A |  |  | 30-Jul-20 | Negative |  | 41.22 | Positive | 2.39 | Positive | 17.93 | Positive | No | N/A |
| 67 | N/A |  |  | 10-Aug-20 | Negative |  | 0.00 | Negative | 0.01 | Negative | 0.08 | Negative | No | N/A |
| 68 | N/A |  |  | 29-Jul-20 | Negative |  | 0.38 | Negative | 0.02 | Negative | 0.07 | Negative | Not reported | N/A |
| 69 | N/A |  |  | 27-Jul-20 | Negative |  | 0.38 | Negative | 0.24 | Negative | 0.07 | Negative | No | N/A |
| 70 | N/A |  |  | 29-Jul-20 | Negative |  | 0.14 | Negative | 0.01 | Negative | 0.06 | Negative | Not reported | N/A |
| 71 | N/A |  |  | 28-Jul-20 | Negative |  | 4.17 | Negative | 0.88 | Negative | 3.01 | Positive | No | N/A |
| 72 | N/A |  |  | 10-Aug-20 | Negative |  | 0.37 | Negative | 0.22 | Negative | 0.08 | Negative | No | N/A |
| 73 | N/A |  |  | 11-Aug-20 | Positive | 17.12 | 0.14 | Negative | 0.00 | Negative | 0.08 | Negative | No | N/A |
| 74 | N/A |  |  | 27-Jul-20 | Negative |  | 0.22 | Negative | 0.69 | Negative | 0.06 | Negative | No | N/A |
| 75 | N/A |  |  | 28-Jul-20 | Negative |  | 42.32 | Positive | 6.43 | Positive | 20.87 | Positive | No | N/A |
| 76 | N/A |  |  | 29-Jul-20 | Positive | 20.6 | 0.00 | Negative | 0.01 | Negative | 0.07 | Negative | No | N/A |
| 77 | N/A |  |  | 29-Jul-20 | Negative |  | 0.00 | Negative | 0.04 | Negative | 0.07 | Negative | No | N/A |
| 78 | N/A |  |  | 21-Aug-20 | Negative |  | 0.30 | Negative | 0.00 | Negative | 0.08 | Negative | No | N/A |
| 79 | N/A |  |  | 26-Jul-20 | Negative |  | 9.49 | Negative | 0.02 | Negative | 0.12 | Negative | Not reported | N/A |
| 80 | N/A |  |  | 23-Jul-20 | Negative |  | 1.71 | Negative | 0.01 | Negative | 0.08 | Negative | No | N/A |
| 81 | N/A |  |  | 11-Aug-20 | Negative |  | 0.00 | Negative | 0.06 | Negative | 0.09 | Negative | No | N/A |
| 82 | N/A |  |  | 10-Aug-20 | Negative |  | 71.76 | Positive | 0.02 | Negative | 0.08 | Negative | No | N/A |
| 83 | N/A |  |  | 29-Jul-20 | Negative |  | 0.15 | Negative | 0.06 | Negative | 0.07 | Negative | No | N/A |
| 84 | N/A |  |  | 10-Aug-20 | Negative |  | 14.52 | Positive | 2.37 | Positive | 6.82 | Positive | No | N/A |
| 85 | N/A |  |  | 29-Jul-20 | Negative |  | 1.90 | Negative | 0.03 | Negative | 0.07 | Negative | No | N/A |
| 86 | N/A |  |  | 10-Aug-20 | Negative |  | 0.23 | Negative | 0.05 | Negative | 0.09 | Negative | No | N/A |
| 87 | N/A |  |  | 10-Aug-20 | Negative |  | 122.32 | Positive | 7.11 | Positive | 120.60 | Positive | No | N/A |
| 88 | N/A |  |  | 29-Jul-20 | Negative |  | 59.19 | Positive | 2.26 | Positive | 80.66 | Positive | No | N/A |
| 89 | N/A |  |  | 27-Jul-20 | Negative |  | 0.69 | Negative | 0.05 | Negative | 0.07 | Negative | No | N/A |
| 90 | N/A |  |  | 10-Aug-20 | Negative |  | 0.37 | Negative | 0.08 | Negative | 0.12 | Negative | No | N/A |
| 91 | N/A |  |  | 29-Jul-20 | Negative |  | 0.14 | Negative | 0.00 | Negative | 0.07 | Negative | No | N/A |
| 92 | N/A |  |  | 29-Jul-20 | Negative |  | 0.17 | Negative | 0.02 | Negative | 0.07 | Negative | No | N/A |
| 93 | N/A |  |  | 28-Jul-20 | Negative |  | 6.24 | Negative | 0.06 | Negative | 0.07 | Negative | No | N/A |
| 94 | N/A |  |  | 10-Aug-20 | Positive | 35.82 | 29.3 | Positive | 2.89 | Positive | 17.90 | Positive | No | N/A |
| 95 | N/A |  |  | 29-Jul-20 | Negative |  | 0.24 | Negative | 0.03 | Negative | 0.07 | Negative | No | N/A |
| 96 | N/A |  |  | 10-Aug-20 | Negative |  | 0.47 | Negative | 0.11 | Negative | 0.09 | Negative | No | N/A |
| 97 | N/A |  |  | 26-Jul-20 | Negative |  | 24.62 | Positive | 6.24 | Positive | 36.56 | Positive | Not reported | N/A |
| 98 | N/A |  |  | 29-Jul-20 | Negative |  | 8.07 | Negative | 0.00 | Negative | 0.07 | Negative | No | N/A |
| 99 | N/A |  |  | 10-Aug-20 | Negative |  | 1.33 | Negative | 0.04 | Negative | 0.10 | Negative | No | N/A |
| 100 | N/A |  |  | 29-Jul-20 | Negative |  | 0.21 | Negative | 0.03 | Negative | 0.07 | Negative | No | N/A |
| 101 | N/A |  |  | 10-Aug-20 | Negative |  | 0.17 | Negative | 0.02 | Negative | 0.10 | Negative | No | N/A |
| 102 | N/A |  |  | 10-Aug-20 | Negative |  | 9.94 | Negative | 0.02 | Negative | 0.10 | Negative | No | N/A |
| 103 | N/A |  |  | 29-Jul-20 | Negative |  | 0.17 | Negative | 0.01 | Negative | 0.08 | Negative | No | N/A |
| 104 | N/A |  |  | 29-Jul-20 | Negative |  | 69.46 | Positive | 1.83 | Positive | 86.12 | Positive | No | N/A |
| 105 | N/A |  |  | 11-Aug-20 | Negative |  | 28.62 | Positive | 19.00 | Positive | 66.20 | Positive | No | N/A |
| 106 | N/A |  |  | 26-Jul-20 | Negative |  | 73.88 | Positive | 6.33 | Positive | 75.37 | Positive | No | N/A |
| 107 | N/A |  |  | 27-Jul-20 | Negative |  | 92.12 | Positive | 11.27 | Positive | 80.42 | Positive | No | N/A |
| 108 | N/A |  |  | 29-Jul-20 | Negative |  | 0.88 | Negative | 0.04 | Negative | 0.07 | Negative | No | N/A |
| 109 | N/A |  |  | 29-Jul-20 | Negative |  | 128.72 | Positive | 24.82 | Positive | 114.60 | Positive | No | N/A |
| 110 | N/A |  |  | 29-Jul-20 | Negative |  | 0.18 | Negative | 0.07 | Negative | 0.07 | Negative | No | N/A |
| 111 | N/A |  |  | 10-Aug-20 | Negative |  | 0.56 | Negative | 0.01 | Negative | 0.08 | Negative | No | N/A |
| 112 | N/A |  |  | 10-Aug-20 | Negative |  | 2.14 | Negative | 0.02 | Negative | 0.08 | Negative | No | N/A |
| 113 | N/A |  |  | 27-Jul-20 | Negative |  | 0.19 | Negative | 0.11 | Negative | 0.07 | Negative | No | N/A |
| 114 | N/A |  |  | 29-Jul-20 | Positive | 21.71 | 0.16 | Negative | 0.19 | Negative | 0.07 | Negative | No | N/A |
| 115 | N/A |  |  | 29-Jul-20 | Negative |  | 34.90 | Positive | 14.3 | Positive | 75.90 | Positive | No | N/A |
| 116 | N/A |  |  | 28-Jul-20 | Negative |  | 66.63 | Positive | 8.60 | Positive | 41.62 | Positive | No | N/A |
| 117 | N/A |  |  | 30-Jul-20 | Negative |  | 494.24 | Positive | 36.29 | Positive | 102.90 | Positive | No | N/A |
| 118 | N/A |  |  | 10-Aug-20 | Positive | 27.73 | 0.39 | Negative | 0.07 | Negative | 0.09 | Negative | No | N/A |
| 119 | N/A |  |  | 27-Jul-20 | Negative |  | 0.19 | Negative | 0.00 | Negative | 0.07 | Negative | No | N/A |
| 120 | N/A |  |  | 10-Aug-20 | Positive | 33.04 | 32.35 | Positive | 2.51 | Positive | 4.20 | Positive | No | N/A |
| 121 | N/A |  |  | 10-Aug-20 | Negative |  | 0.21 | Negative | 0.01 | Negative | 0.09 | Negative | No | N/A |
| 122 | N/A |  |  | 23-Jul-20 | Negative |  | 5.35 | Negative | 0.05 | Negative | 0.07 | Negative | No | N/A |
| 123 | N/A |  |  | 27-Jul-20 | Negative |  | 0.45 | Negative | 0.02 | Negative | 0.07 | Negative | No | N/A |
| 124 | N/A |  |  | 29-Jul-20 | Negative |  | 27.54 | Positive | 4.35 | Positive | 1.78 | Positive | No | N/A |
| 125 | N/A |  |  | 10-Aug-20 | Negative |  | 18.40 | Positive | 1.09 | Positive | 33.52 | Positive | No | N/A |
| 126 | N/A |  |  | 10-Aug-20 | Positive | 26.95 | 10.50 | Positive | 0.15 | Negative | 0.18 | Negative | No | N/A |
| 127 | N/A |  |  | 10-Aug-20 | Positive | 18.86 | 1.01 | Negative | 0.01 | Negative | 0.08 | Negative | No | N/A |
| 128 | N/A |  |  | 10-Aug-20 | Positive | 33.81 | 220.63 | Positive | 0.62 | Negative | 7.72 | Positive | No | N/A |
| 129 | N/A |  |  | 10-Aug-20 | Negative |  | 16.60 | Positive | 0.07 | Negative | 0.08 | Negative | No | N/A |
| 130 | N/A |  |  | 27-Jul-20 | Negative |  | 120.22 | Positive | 5.59 | Positive | 128.90 | Positive | No | N/A |
| 131 | N/A |  |  | 13-Aug-20 | Positive | 28.32 | 36.22 | Positive | 0.46 | Negative | 9.23 | Positive | No | N/A |
| 132 | N/A |  |  | 10-Aug-20 | Negative |  | 0.24 | Negative | 0.01 | Negative | 0.08 | Negative | No | N/A |
| 133 | N/A |  |  | 29-Jul-20 | Negative |  | 0.32 | Negative | 0.02 | Negative | 0.07 | Negative | No | N/A |
| 134 | N/A |  |  | 23-Jul-20 | Negative |  | 32.04 | Positive | 1.12 | Positive | 23.86 | Positive | No | N/A |
| 135 | N/A |  |  | 29-Jul-20 | Negative |  | 133.57 | Positive | 1.73 | Positive | 102.50 | Positive | No | N/A |
| 136 | N/A |  |  | 27-Jul-20 | Negative |  | 137.99 | Positive | 5.45 | Positive | 80.79 | Positive | No | N/A |
| 137 | N/A |  |  | 10-Aug-20 | Negative |  | 4.72 | Negative | 0.46 | Negative | 10.98 | Positive | No | N/A |
| 138 | N/A |  |  | 10-Aug-20 | Negative |  | 0.14 | Negative | 0.05 | Negative | 0.08 | Negative | No | N/A |
| 139 | N/A |  |  | 28-Jul-20 | Negative |  | 59.00 | Positive | 2.49 | Positive | 33.77 | Positive | No | N/A |
| 140 | N/A |  |  | 10-Aug-20 | Positive | 23.05 | 0.21 | Negative | 0.21 | Negative | 0.08 | Negative | No | N/A |
| 141 | N/A |  |  | 10-Aug-20 | Negative |  | 2.12 | Negative | 0.01 | Negative | 0.08 | Negative | No | N/A |
| 142 | N/A |  |  | 10-Aug-20 | Negative |  | 43.84 | Positive | 5.65 | Positive | 28.51 | Positive | No | N/A |
| 143 | N/A |  |  | 10-Aug-20 | Negative |  | 0.87 | Negative | 0.05 | Negative | 0.08 | Negative | No | N/A |
| 144 | N/A |  |  | 27-Jul-20 | Negative |  | 0.15 | Negative | 0.02 | Negative | 0.07 | Negative | No | N/A |
| 145 | N/A |  |  | 27-Jul-20 | Negative |  | 84.86 | Positive | 24.3 | Positive | 31.19 | Positive | No | N/A |
| 146 | N/A |  |  | 10-Aug-20 | Negative |  | 0.29 | Negative | 0.04 | Negative | 0.10 | Negative | No | N/A |
| 147 | N/A |  |  | 10-Aug-20 | Negative |  | 39.57 | Positive | 0.94 | Negative | 10.92 | Positive | No | N/A |
| 148 | N/A |  |  | 10-Aug-20 | Negative |  | 444.57 | Positive | 20.31 | Positive | 74.54 | Positive | No | N/A |
| 149 | N/A |  |  | 10-Aug-20 | Negative |  | 81.69 | Positive | 25.89 | Positive | 59.53 | Positive | Symptomatic | N/A |
| 150 | N/A |  |  | 10-Aug-20 | Negative |  | 0.00 | Negative | 0.01 | Negative | 0.09 | Negative | No | N/A |
| 151 | N/A |  |  | 31-Jul-20 | Positive | 18.7 | 0.27 | Negative | 0.03 | Negative | 0.12 | Negative | No | N/A |
| 152 | N/A |  |  | 10-Aug-20 | Positive | 19.5 | 0.36 | Negative | 0.07 | Negative | 0.08 | Negative | No | N/A |
| 153 | N/A |  |  | 23-Jul-20 | Negative |  | 0.34 | Negative | 0.04 | Negative | 0.07 | Negative | No | N/A |
| 154 | N/A |  |  | 29-Jul-20 | Positive | 17.6 | 0.18 | Negative | 0.07 | Negative | 0.07 | Negative | No | N/A |
| 155 | N/A |  |  | 29-Jul-20 | Negative |  | 0.00 | Negative | 0.51 | Negative | 0.07 | Negative | No | N/A |
| 156 | N/A |  |  | 29-Jul-20 | Negative |  | 0.34 | Negative | 0.01 | Negative | 0.07 | Negative | No | N/A |
| 157 | N/A |  |  | 29-Jul-20 | Negative |  | 0.00 | Negative | 0.00 | Negative | 0.07 | Negative | No | N/A |
| 158 | N/A |  |  | 10-Aug-20 | Negative |  | 10.84 | Positive | 1.66 | Positive | 1.57 | Positive | No | N/A |
| 159 | N/A |  |  | 10-Aug-20 | Negative |  | 3.14 | Negative | 0.00 | Negative | 0.09 | Negative | No | N/A |
| 160 | N/A |  |  | 28-Jul-20 | Negative |  | 0.47 | Negative | 0.23 | Negative | 0.07 | Negative | No | N/A |
| 161 | N/A |  |  | 29-Jul-20 | Negative |  | 6.67 | Negative | 0.07 | Negative | 0.07 | Negative | No | N/A |
| 162 | N/A |  |  | 29-Jul-20 | Negative |  | 0.23 | Negative | 0.10 | Negative | 0.07 | Negative | No | N/A |
| 163 | N/A |  |  | 10-Aug-20 | Negative |  | 7.78 | Negative | 0.04 | Negative | 0.09 | Negative | No | N/A |
| 164 | N/A |  |  | 10-Aug-20 | Negative |  | 0.58 | Negative | 0.11 | Negative | 0.08 | Negative | No | N/A |
| 165 | N/A |  |  | 10-Aug-20 | Negative |  | 24.89 | Positive | 1.33 | Positive | 57.9 | Positive | No | N/A |
| 166 | N/A |  |  | 29-Jul-20 | Positive | 20.89 | 0.29 | Negative | 0.03 | Negative | 0.07 | Negative | No | N/A |
| 167 | N/A |  |  | 10-Aug-20 | Negative |  | 23.67 | Positive | 2.56 | Positive | 24.40 | Positive | No | N/A |
| 168 | N/A |  |  | 10-Aug-20 | Negative |  | 213.1 | Positive | 2.78 | Positive | 91.42 | Positive | No | N/A |
| 169 | N/A |  |  | 10-Aug-20 | Negative |  | 0.19 | Negative | 0.01 | Negative | 0.09 | Negative | No | N/A |
| 170 | N/A |  |  | 29-Jul-20 | Negative |  | 6.71 | Negative | 0.03 | Negative | 0.07 | Negative | No | N/A |
| 171 | N/A |  |  | 29-Jul-20 | Negative |  | 61.68 | Positive | 14.62 | Positive | 75.12 | Positive | No | N/A |
| 172 | N/A |  |  | 10-Aug-20 | Negative |  | 0.66 | Negative | 0.02 | Negative | 0.08 | Negative | No | N/A |
| 173 | N/A |  |  | 10-Aug-20 | Negative |  | 1.52 | Negative | 0.00 | Negative | 0.09 | Negative | No | N/A |
| 174 | N/A |  |  | 29-Jul-20 | Positive | 24.09 | 12.05 | Positive | 1.29 | Positive | 0.12 | Negative | No | N/A |
| 175 | N/A |  |  | 23-Jul-20 | Negative |  | 0.48 | Negative | 0.03 | Negative | 0.07 | Negative | No | N/A |
| 176 | N/A |  |  | 27-Jul-20 | Negative |  | 0.25 | Negative | 0.00 | Negative | 0.07 | Negative | No | N/A |
| 177 | N/A |  |  | 10-Aug-20 | Negative |  | 0.23 | Negative | 0.17 | Negative | 0.08 | Negative | No | N/A |
| 178 | N/A |  |  | 10-Aug-20 | Positive | 35.93 | 226.94 | Positive | 4.01 | Positive | 22.99 | Positive | No | N/A |
| 179 | N/A |  |  | 10-Aug-20 | Negative |  | 243.66 | Positive | 2.77 | Positive | 39.02 | Positive | No | N/A |
| 180 | N/A |  |  | 29-Jul-20 | Positive | 27.97 | 0.21 | Negative | 0.82 | Negative | 0.07 | Negative | No | N/A |
| 181 | N/A |  |  | 26-Jul-20 | Negative |  | 27.55 | Positive | 1.29 | Positive | 60.20 | Positive | No | N/A |
| 182 | N/A |  |  | 29-Jul-20 | Negative |  | 11.22 | Positive | 0.35 | Negative | 0.07 | Negative | Not reported | N/A |
| 183 | N/A |  |  | 10-Aug-20 | Negative |  | 45.31 | Positive | 5.61 | Positive | 27.47 | Positive | No | N/A |
| 184 | N/A |  |  | 23-Jul-20 | Negative |  | 7.90 | Negative | 0.01 | Negative | 0.07 | Negative | No | N/A |
| 185 | N/A |  |  | 10-Aug-20 | Negative |  | 103.00 | Positive | 8.41 | Positive | 47.13 | Positive | No | N/A |
| 186 | N/A |  |  | 27-Jul-20 | Negative |  | 225.81 | Positive | 25.73 | Positive | 112.10 | Positive | No | N/A |
| 187 | N/A |  |  | 10-Aug-20 | Positive | 34.33 | 140.59 | Positive | 29.8 | Positive | 87.85 | Positive | No | N/A |
| 188 | N/A |  |  | 26-Jul-20 | Negative |  | 29.54 | Positive | 13.75 | Positive | 14.55 | Positive | Not reported | N/A |
| 189 | N/A |  |  | 10-Aug-20 | Negative |  | 5.04 | Negative | 0.04 | Negative | 0.10 | Negative | No | N/A |
| 190 | N/A |  |  | 28-Jul-20 | Negative |  | 184.14 | Positive | 9.25 | Positive | 98.72 | Positive | No | N/A |
| 191 | N/A |  |  | 10-Aug-20 | Negative |  | 7.87 | Negative | 1.75 | Positive | 8.41 | Positive | No | N/A |
| 192 | N/A |  |  | 29-Jul-20 | Negative |  | 0.00 | Negative | 0.05 | Negative | 0.07 | Negative | No | N/A |
| 193 | N/A |  |  | 10-Aug-20 | Positive | 23.48 | 0.23 | Negative | 0.03 | Negative | 0.09 | Negative | No | N/A |
| 194 | N/A |  |  | 29-Jul-20 | Negative |  | 0.79 | Negative | 0.02 | Negative | 0.07 | Negative | No | N/A |
| 195 | N/A |  |  | 10-Aug-20 | Negative |  | 0.26 | Negative | 0.00 | Negative | 0.09 | Negative | No | N/A |
| 196 | N/A |  |  | 11-Aug-20 | Positive | 17.89 | 0.14 | Negative | 0.01 | Negative | 0.08 | Negative | No | N/A |
| 197 | N/A |  |  | 10-Aug-20 | Negative |  | 2.41 | Negative | 0.06 | Negative | 0.19 | Negative | No | N/A |
| 198 | N/A |  |  | 10-Aug-20 | Negative |  | 0.33 | Negative | 0.19 | Negative | 0.09 | Negative | No | N/A |
| 199 | N/A |  |  | 26-Jul-20 | Negative |  | 127.57 | Positive | 21.14 | Positive | 133.10 | Positive | No | N/A |
| 200 | N/A |  |  | 29-Jul-20 | Negative |  | 0.14 | Negative | 0.02 | Negative | 0.07 | Negative | No | N/A |
| 201 | N/A |  |  | 29-Jul-20 | Negative |  | 0.42 | Negative | 0.00 | Negative | 0.07 | Negative | No | N/A |
| 202 | N/A |  |  | 29-Jul-20 | Negative |  | 0.34 | Negative | 0.05 | Negative | 0.07 | Negative | No | N/A |
| 203 | N/A |  |  | 10-Aug-20 | Negative |  | 216.24 | Positive | 9.75 | Positive | 92.44 | Positive | No | N/A |
| 204 | N/A |  |  | 10-Aug-20 | Negative |  | 0.39 | Negative | 0.02 | Negative | 0.09 | Negative | No | N/A |
| 205 | N/A |  |  | 10-Aug-20 | Negative |  | 60.44 | Positive | 2.38 | Positive | 16.19 | Positive | No | N/A |
| 206 | N/A |  |  | 29-Jul-20 | Negative |  | 0.36 | Negative | 0.17 | Negative | 0.07 | Negative | No | N/A |
| 207 | N/A |  |  | 29-Jul-20 | Positive | 34.65 | 3.19 | Negative | 0.01 | Negative | 0.07 | Negative | No | N/A |
| 208 | N/A |  |  | 29-Jul-20 | Negative |  | 1.84 | Negative | 0.01 | Negative | 0.07 | Negative | No | N/A |
| 209 | N/A |  |  | 29-Jul-20 | Negative |  | 0.21 | Negative | 0.06 | Negative | 0.07 | Negative | No | N/A |
| 210 | N/A |  |  | 10-Aug-20 | Negative |  | 1.61 | Negative | 0.13 | Negative | 0.09 | Negative | No | N/A |
| 211 | N/A |  |  | 10-Aug-20 | Negative |  | 0.25 | Negative | 0.02 | Negative | 0.09 | Negative | No | N/A |
| 212 | N/A |  |  | 29-Jul-20 | Positive | 33.01 | 52.16 | Positive | 22.91 | Positive | 9.26 | Positive | No | N/A |
| 213 | N/A |  |  | 29-Jul-20 | Negative |  | 140.62 | Positive | 0.00 | Negative | 0.11 | Negative | No | N/A |
| 214 | N/A |  |  | 29-Jul-20 | Negative |  | 35.94 | Positive | 2.29 | Positive | 28.93 | Positive | No | N/A |
| 215 | N/A |  |  | 10-Aug-20 | Negative |  | 57.61 | Positive | 2.01 | Positive | 72.92 | Positive | No | N/A |
| 216 | N/A |  |  | 10-Aug-20 | Negative |  | 22.18 | Positive | 3.89 | Positive | 40.02 | Positive | No | N/A |
| 217 | N/A |  |  | 10-Aug-20 | Negative |  | 37.27 | Positive | 4.65 | Positive | 10.10 | Positive | No | N/A |
| 218 | N/A |  |  | 10-Aug-20 | Negative |  | 1.54 | Negative | 0.08 | Negative | 0.09 | Negative | No | N/A |
| 219 | N/A |  |  | 29-Jul-20 | Positive | 32.31 | 1.36 | Negative | 0.12 | Negative | 0.07 | Negative | No | N/A |
| 220 | N/A |  |  | 10-Aug-20 | Negative |  | 48.51 | Positive | 5.12 | Positive | 131.90 | Positive | No | N/A |
| 221 | N/A |  |  | 26-Jul-20 | Negative |  | 0.18 | Negative | 0.09 | Negative | 0.07 | Negative | No | N/A |
| 222 | N/A |  |  | 29-Jul-20 | Negative |  | 0.48 | Negative | 0.00 | Negative | 0.07 | Negative | No | N/A |
| 223 | N/A |  |  | 10-Aug-20 | Negative |  | 7.29 | Negative | 4.73 | Positive | 7.35 | Positive | No | N/A |
| 224 | N/A |  |  | 29-Jul-20 | Positive | 21.66 | 0.70 | Negative | 0.08 | Negative | 0.07 | Negative | No | N/A |
| 225 | N/A |  |  | 29-Jul-20 | Positive | 15.96 | 0.17 | Negative | 0.01 | Negative | 0.07 | Negative | No | N/A |
| 226 | N/A |  |  | 29-Jul-20 | Negative |  | 0.26 | Negative | 0.07 | Negative | 0.07 | Negative | No | N/A |
| 227 | N/A |  |  | 27-Jul-20 | Negative |  | 0.25 | Negative | 0.02 | Negative | 0.07 | Negative | No | N/A |
| 228 | N/A |  |  | 10-Aug-20 | Negative |  | 0.17 | Negative | 5.56 | Positive | 40.35 | Positive | No | N/A |
| 229 | N/A |  |  | 10-Aug-20 | Negative |  | 0.28 | Negative | 0.08 | Negative | 0.10 | Negative | No | N/A |
| 230 | N/A |  |  | 29-Jul-20 | Negative |  | 0.16 | Negative | 0.08 | Negative | 0.07 | Negative | No | N/A |
| 231 | N/A |  |  | 29-Jul-20 | Negative |  | 14.39 | Positive | 3.83 | Positive | 19.30 | Positive | No | N/A |
| 232 | N/A |  |  | 29-Jul-20 | Positive | 23.89 | 16.48 | Positive | 0.19 | Negative | 0.09 | Negative | No | N/A |
| 233 | N/A |  |  | 10-Aug-20 | Negative |  | 135.98 | Positive | 12.80 | Positive | 100.70 | Positive | No | N/A |
| 234 | N/A |  |  | 27-Jul-20 | Negative |  | 639.70 | Positive | 11.01 | Positive | 93.93 | Positive | No | N/A |
| 235 | N/A |  |  | 10-Aug-20 | Negative |  | 0.23 | Negative | 0.09 | Negative | 0.09 | Negative | No | N/A |
| 236 | N/A |  |  | 23-Jul-20 | Negative |  | 0.16 | Negative | 0.09 | Negative | 0.07 | Negative | No | N/A |
| 237 | N/A |  |  | 29-Jul-20 | Negative |  | 1.90 | Negative | 0.01 | Negative | 0.07 | Negative | No | N/A |
| 238 | N/A |  |  | 10-Aug-20 | Negative |  | 0.18 | Negative | 0.04 | Negative | 0.08 | Negative | No | N/A |
| 239 | N/A |  |  | 23-Jul-20 | Negative |  | 1.19 | Negative | 0.06 | Negative | 0.07 | Negative | No | N/A |
| 240 | N/A |  |  | 27-Jul-20 | Negative |  | 0.51 | Negative | 0.08 | Negative | 0.07 | Negative | No | N/A |
| 241 | N/A |  |  | 29-Jul-20 | Negative |  | 0.22 | Negative | 0.01 | Negative | 0.07 | Negative | No | N/A |
| 242 | N/A |  |  | 27-Jul-20 | Negative |  | 25.66 | Positive | 1.79 | Positive | 24.33 | Positive | No | N/A |
| 243 | N/A |  |  | 29-Jul-20 | Negative |  | 0.46 | Negative | 0.03 | Negative | 0.07 | Negative | No | N/A |
| 244 | N/A |  |  | 10-Aug-20 | Negative |  | 0.27 | Negative | 0.10 | Negative | 0.09 | Negative | No | N/A |
| 245 | N/A |  |  | 10-Aug-20 | Negative |  | 41.44 | Positive | 3.44 | Positive | 132.30 | Positive | No | N/A |
| 246 | N/A |  |  | 10-Aug-20 | Negative |  | 5.60 | Negative | 1.37 | Positive | 6.98 | Positive | No | N/A |
| 247 | N/A |  |  | 27-Jul-20 | Negative |  | 0.17 | Negative | 0.11 | Negative | 0.07 | Negative | No | N/A |
| 248 | N/A |  |  | 27-Jul-20 | Negative |  | 155.03 | Positive | 8.41 | Positive | 69.92 | Positive | No | N/A |
| 249 | N/A |  |  | 10-Aug-20 | Negative |  | 27.43 | Positive | 1.64 | Positive | 7.38 | Positive | No | N/A |
| 250 | N/A |  |  | 29-Jul-20 | Negative |  | 3.75 | Negative | 0.13 | Negative | 0.07 | Negative | No | N/A |
| 251 | N/A |  |  | 29-Jul-20 | Negative |  | 0.55 | Negative | 0.00 | Negative | 0.07 | Negative | No | N/A |
| 252 | N/A |  |  | 27-Jul-20 | Negative |  | 0.00 | Negative | 0.01 | Negative | 0.07 | Negative | No | N/A |
| 253 | N/A |  |  | 27-Jul-20 | Negative |  | 0.00 | Negative | 0.06 | Negative | 0.07 | Negative | No | N/A |
| 254 | N/A |  |  | 10-Aug-20 | Positive | 20.61 | 1.41 | Negative | 0.04 | Negative | 0.09 | Negative | No | N/A |
| 255 | N/A |  |  | 27-Jul-20 | Negative |  | 8.06 | Negative | 1.09 | Positive | 10.18 | Positive | No | N/A |
| 256 | N/A |  |  | 29-Jul-20 | Negative |  | 87.08 | Positive | 4.41 | Positive | 81.39 | Positive | No | N/A |
| 257 | N/A |  |  | 26-Jul-20 | Negative |  | 0.25 | Negative | 0.01 | Negative | 0.07 | Negative | No | N/A |
| 258 | N/A |  |  | 10-Aug-20 | Negative |  | 0.34 | Negative | 0.05 | Negative | 0.09 | Negative | No | N/A |
| 259 | N/A |  |  | 29-Jul-20 | Negative |  | 0.26 | Negative | 0.04 | Negative | 0.07 | Negative | No | N/A |
| 260 | N/A |  |  | 26-Jul-20 | Negative |  | 19.11 | Positive | 9.37 | Positive | 13.19 | Positive | No | N/A |
| 261 | N/A |  |  | 29-Jul-20 | Negative |  | 1.96 | Negative | 0.03 | Negative | 0.07 | Negative | No | N/A |
| 262 | N/A |  |  | 29-Jul-20 | Negative |  | 2.44 | Negative | 0.03 | Negative | 0.08 | Negative | No | N/A |
| 263 | N/A |  |  | 10-Aug-20 | Negative |  | 187.18 | Positive | 22.08 | Positive | 99.68 | Positive | No | N/A |
| 264 | N/A |  |  | 10-Aug-20 | Negative |  | 72.45 | Positive | 37.74 | Positive | 31.75 | Positive | No | N/A |
| 265 | N/A |  |  | 10-Aug-20 | Negative |  | 79.20 | Positive | 8.41 | Positive | 26.50 | Positive | No | N/A |
| 266 | N/A |  |  | 29-Jul-20 | Negative |  | 62.69 | Positive | 10.34 | Positive | 69.28 | Positive | No | N/A |
| 267 | N/A |  |  | 27-Jul-20 | Negative |  | 0.26 | Negative | 0.12 | Negative | 0.07 | Negative | No | N/A |
| 268 | N/A |  |  | 10-Aug-20 | Negative |  | 0.00 | Negative | 0.03 | Negative | 0.09 | Negative | No | N/A |
| 269 | N/A |  |  | 29-Jul-20 | Negative |  | 0.54 | Negative | 0.01 | Negative | 0.07 | Negative | No | N/A |
| 270 | N/A |  |  | 29-Jul-20 | Negative |  | 31.29 | Positive | 7.55 | Positive | 19.71 | Positive | No | N/A |
| 271 | N/A |  |  | 29-Jul-20 | Negative |  | 21.61 | Positive | 2.34 | Positive | 85.35 | Positive | No | N/A |
| 272 | N/A |  |  | 10-Aug-20 | Negative |  | 311.02 | Positive | 4.06 | Positive | 100.40 | Positive | No | N/A |
| 273 | N/A |  |  | 29-Jul-20 | Negative |  | 11.77 | Positive | 2.41 | Positive | 1.60 | Positive | No | N/A |
| 274 | N/A |  |  | 29-Jul-20 | Negative |  | 0.21 | Negative | 0.25 | Negative | 0.07 | Negative | No | N/A |
| 275 | N/A |  |  | 7-Aug-20 | Positive | 28.76 | 0.32 | Negative | 0.11 | Negative | 0.11 | Negative | No | N/A |
| 276 | N/A |  |  | 29-Jul-20 | Negative |  | 29.21 | Positive | 2.75 | Positive | 3.54 | Positive | No | N/A |
| 277 | N/A |  |  | 29-Jul-20 | Negative |  | 159.77 | Positive | 21.92 | Positive | 6.24 | Positive | No | N/A |
| 278 | N/A |  |  | 27-Jul-20 | Negative |  | 0.37 | Negative | 0.04 | Negative | 0.07 | Negative | No | N/A |
| 279 | N/A |  |  | 10-Aug-20 | Negative |  | 0.45 | Negative | 0.12 | Negative | 0.09 | Negative | No | N/A |
| 280 | N/A |  |  | 29-Jul-20 | Positive | 32.34 | 20.61 | Positive | 2.30 | Positive | 0.51 | Negative | No | N/A |
| 281 | N/A |  |  | 10-Aug-20 | Negative |  | 25.03 | Positive | 9.61 | Positive | 63.81 | Positive | No | N/A |
| 282 | N/A |  |  | 10-Aug-20 | Negative |  | 18.49 | Positive | 7.86 | Positive | 78.13 | Positive | No | N/A |
| 283 | N/A |  |  | 10-Aug-20 | Positive | 38.94 | 185.98 | Positive | 8.02 | Positive | 46.09 | Positive | No | N/A |
| 284 | N/A |  |  | 10-Aug-20 | Positive | 23.04 | 1.17 | Negative | 0.02 | Negative | 0.10 | Negative | No | N/A |
| 285 | N/A |  |  | 10-Aug-20 | Negative |  | 0.22 | Negative | 0.04 | Negative | 0.09 | Negative | No | N/A |
| 286 | N/A |  |  | 29-Jul-20 | Negative |  | 0.00 | Negative | 0.04 | Negative | 0.07 | Negative | No | N/A |
| 287 | N/A |  |  | 31-Aug-20 | Negative |  | 0.33 | Negative | 0.03 | Negative | 0.09 | Negative | No | N/A |
| 288 | N/A |  |  | 10-Aug-20 | Negative |  | 16.67 | Positive | 2.55 | Positive | 6.96 | Positive | No | N/A |
| 289 | N/A |  |  | 17-Aug-20 | Negative |  | 1.01 | Negative | 0.01 | Negative | 0.09 | Negative | No | N/A |
| 290 | N/A |  |  | 27-Jul-20 | Negative |  | 25.18 | Positive | 13.43 | Positive | 44.56 | Positive | No | N/A |
| 291 | N/A |  |  | 27-Jul-20 | Negative |  | 101.96 | Positive | 8.80 | Positive | 102.20 | Positive | No | N/A |
| 292 | N/A |  |  | 27-Jul-20 | Negative |  | 0.00 | Negative | 0.05 | Negative | 0.07 | Negative | No | N/A |
| 293 | N/A |  |  | 27-Jul-20 | Negative |  | 166.18 | Positive | 12.70 | Positive | 119.00 | Positive | No | N/A |
| 294 | N/A |  |  | 27-Jul-20 | Negative |  | 1.29 | Negative | 0.11 | Negative | 0.13 | Negative | No | N/A |
| 295 | N/A |  |  | 27-Jul-20 | Negative |  | 0.28 | Negative | 0.03 | Negative | 0.07 | Negative | No | N/A |
| 296 | N/A |  |  | 27-Jul-20 | Negative |  | 15.7 | Positive | 0.04 | Negative | 0.15 | Negative | No | N/A |
| 297 | N/A |  |  | 29-Jul-20 | Negative |  | 0.19 | Negative | 0.02 | Negative | 0.07 | Negative | No | N/A |
| 298 | N/A |  |  | 29-Jul-20 | Negative |  | 17.17 | Positive | 1.01 | Positive | 22.59 | Positive | No | N/A |
| 299 | N/A |  |  | 29-Jul-20 | Negative |  | 0.97 | Negative | 0.00 | Negative | 0.07 | Negative | No | N/A |
| 300 | N/A |  |  | 29-Jul-20 | Negative |  | 279.51 | Positive | 12.39 | Positive | 67.95 | Positive | No | N/A |
| 301 | N/A |  |  | 29-Jul-20 | Negative |  | 0.41 | Negative | 0.00 | Negative | 0.07 | Negative | No | N/A |
| 302 | N/A |  |  | 29-Jul-20 | Negative |  | 1.15 | Negative | 0.00 | Negative | 0.07 | Negative | No | N/A |
| 303 | N/A |  |  | 29-Jul-20 | Negative |  | 50.65 | Positive | 16.56 | Positive | 74.14 | Positive | No | N/A |
| 304 | N/A |  |  | 29-Jul-20 | Negative |  | 1.48 | Negative | 0.03 | Negative | 0.08 | Negative | No | N/A |
| 305 | N/A |  |  | 29-Jul-20 | Negative |  | 0.84 | Negative | 0.10 | Negative | 0.07 | Negative | No | N/A |
| 306 | N/A |  |  | 29-Jul-20 | Negative |  | 88.35 | Positive | 19.78 | Positive | 75.56 | Positive | No | N/A |
| 307 | N/A |  |  | 29-Jul-20 | Negative |  | 27.67 | Positive | 15.35 | Positive | 55.25 | Positive | No | N/A |
| 308 | N/A |  |  | 29-Jul-20 | Negative |  | 75.5 | Positive | 4.83 | Positive | 127.5 | Positive | No | N/A |
| 309 | N/A |  |  | 29-Jul-20 | Negative |  | 67.37 | Positive | 4.24 | Positive | 27.52 | Positive | No | N/A |
| 310 | N/A |  |  | 29-Jul-20 | Negative |  | 1.12 | Negative | 0.06 | Negative | 0.07 | Negative | No | N/A |
| 311 | N/A |  |  | 29-Jul-20 | Negative |  | 1.74 | Negative | 0.01 | Negative | 0.07 | Negative | No | N/A |
| 312 | N/A |  |  | 29-Jul-20 | Negative |  | 0.00 | Negative | 0.00 | Negative | 0.07 | Negative | No | N/A |
| 313 | N/A |  |  | 29-Jul-20 | Negative |  | 0.15 | Negative | 0.04 | Negative | 0.07 | Negative | No | N/A |
| 314 | N/A |  |  | 29-Jul-20 | Negative |  | 112.5 | Positive | 21.14 | Positive | 47.52 | Positive | No | N/A |
| 315 | N/A |  |  | 29-Jul-20 | Negative |  | 202.17 | Positive | 11.73 | Positive | 120.10 | Positive | No | N/A |
| 316 | N/A |  |  | 29-Jul-20 | Negative |  | 11.76 | Positive | 2.29 | Positive | 13.67 | Positive | No | N/A |
| 317 | N/A |  |  | 29-Jul-20 | Negative |  | 0.25 | Negative | 0.01 | Negative | 0.06 | Negative | No | N/A |
| 318 | N/A |  |  | 29-Jul-20 | Negative |  | 0.25 | Negative | 0.02 | Negative | 0.07 | Negative | No | N/A |
| 319 | N/A |  |  | 29-Jul-20 | Negative |  | 4.07 | Negative | 2.48 | Positive | 1.16 | Positive | No | N/A |
| 320 | N/A |  |  | 29-Jul-20 | Negative |  | 5.46 | Negative | 0.05 | Negative | 0.19 | Negative | No | N/A |
| 321 | N/A |  |  | 29-Jul-20 | Negative |  | 0.82 | Negative | 0.03 | Negative | 0.07 | Negative | No | N/A |
| 322 | N/A |  |  | 29-Jul-20 | Negative |  | 101.49 | Positive | 13.20 | Positive | 28.93 | Positive | No | N/A |
| 323 | N/A |  |  | 29-Jul-20 | Negative |  | 16.81 | Positive | 4.89 | Positive | 18.44 | Positive | No | N/A |
| 324 | N/A |  |  | 29-Jul-20 | Negative |  | 42.28 | Positive | 9.41 | Positive | 18.39 | Positive | No | N/A |
| 325 | N/A |  |  | 29-Jul-20 | Negative |  | 156.99 | Positive | 20.00 | Positive | 44.38 | Positive | No | N/A |
| 326 | N/A |  |  | 29-Jul-20 | Negative |  | 0.16 | Negative | 0.01 | Negative | 0.07 | Negative | No | N/A |
| 327 | N/A |  |  | 29-Jul-20 | Negative |  | 165.3 | Positive | 14.41 | Positive | 20.85 | Positive | No | N/A |
| 328 | N/A |  |  | 29-Jul-20 | Negative |  | 6.41 | Negative | 0.68 | Negative | 2.59 | Positive | No | N/A |
| 329 | N/A |  |  | 29-Jul-20 | Negative |  | 0.31 | Negative | 0.10 | Negative | 0.08 | Negative | No | N/A |
| 330 | N/A |  |  | 29-Jul-20 | Positive | 33.91 | 0.25 | Negative | 0.00 | Negative | 0.07 | Negative | No | N/A |
| 331 | N/A |  |  | 29-Jul-20 | Negative |  | 121.23 | Positive | 21.48 | Positive | 134.10 | Positive | No | N/A |
| 332 | N/A |  |  | 29-Jul-20 | Negative |  | 0.42 | Negative | 0.02 | Negative | 0.07 | Negative | No | N/A |
| 333 | N/A |  |  | 29-Jul-20 | Negative |  | 1.96 | Negative | 0.02 | Negative | 0.08 | Negative | No | N/A |
| 334 | N/A |  |  | 29-Jul-20 | Negative |  | 4.04 | Negative | 0.05 | Negative | 0.06 | Negative | No | N/A |
| 335 | N/A |  |  | 29-Jul-20 | Negative |  | 0.23 | Negative | 0.15 | Negative | 0.07 | Negative | No | N/A |
| 336 | N/A |  |  | 29-Jul-20 | Negative |  | 34.18 | Positive | 20.02 | Positive | 44.96 | Positive | No | N/A |
| 337 | N/A |  |  | 29-Jul-20 | Negative |  | 80.22 | Positive | 0.86 | Negative | 5.62 | Positive | No | N/A |
| 338 | N/A |  |  | 29-Jul-20 | Negative |  | 23.62 | Positive | 10.85 | Positive | 11.38 | Positive | No | N/A |
| 339 | N/A |  |  | 29-Jul-20 | Negative |  | 21.54 | Positive | 2.44 | Positive | 29.19 | Positive | No | N/A |
| 340 | N/A |  |  | 29-Jul-20 | Negative |  | 0.16 | Negative | 0.02 | Negative | 0.07 | Negative | No | N/A |
| 341 | N/A |  |  | 29-Jul-20 | Negative |  | 0.31 | Negative | 0.28 | Negative | 0.07 | Negative | No | N/A |
| 342 | N/A |  |  | 29-Jul-20 | Negative |  | 0.19 | Negative | 0.00 | Negative | 0.07 | Negative | No | N/A |
| 343 | N/A |  |  | 29-Jul-20 | Negative |  | 74.29 | Positive | 5.55 | Positive | 50.58 | Positive | No | N/A |
| 344 | N/A |  |  | 29-Jul-20 | Negative |  | 0.36 | Negative | 0.03 | Negative | 0.07 | Negative | No | N/A |
| 345 | N/A |  |  | 29-Jul-20 | Negative |  | 61.72 | Positive | 3.40 | Positive | 34.03 | Positive | No | N/A |
| 346 | N/A |  |  | 29-Jul-20 | Negative |  | 40.59 | Positive | 14.82 | Positive | 63.25 | Positive | No | N/A |
| 347 | N/A |  |  | 29-Jul-20 | Negative |  | 0.00 | Negative | 0.00 | Negative | 0.07 | Negative | No | N/A |
| 348 | N/A |  |  | 29-Jul-20 | Negative |  | 0.22 | Negative | 0.22 | Negative | 0.07 | Negative | No | N/A |
| 349 | N/A |  |  | 29-Jul-20 | Negative |  | 54.71 | Positive | 7.24 | Positive | 1.66 | Positive | No | N/A |
| 350 | N/A |  |  | 29-Jul-20 | Negative |  | 4.28 | Negative | 0.00 | Negative | 0.07 | Negative | No | N/A |
| 351 | N/A |  |  | 29-Jul-20 | Negative |  | 0.14 | Negative | 0.02 | Negative | 0.07 | Negative | No | N/A |
| 352 | N/A |  |  | 29-Jul-20 | Positive | 26.21 | 4.53 | Negative | 0.07 | Negative | 0.37 | Negative | No | N/A |
| 353 | N/A |  |  | 29-Jul-20 | Negative |  | 2.36 | Negative | 0.00 | Negative | 0.07 | Negative | No | N/A |
| 354 | N/A |  |  | 29-Jul-20 | Negative |  | 0.22 | Negative | 0.05 | Negative | 0.07 | Negative | No | N/A |
| 355 | N/A |  |  | 29-Jul-20 | Positive | 29.18 | 2.36 | Negative | 2.11 | Positive | 0.10 | Negative | No | N/A |
| 356 | N/A |  |  | 29-Jul-20 | Negative |  | 0.41 | Negative | 0.08 | Negative | 0.07 | Negative | No | N/A |
| 357 | N/A |  |  | 29-Jul-20 | Negative |  | 10.49 | Positive | 3.34 | Positive | 0.73 | Negative | No | N/A |
| 358 | N/A |  |  | 10-Aug-20 | Negative |  | 0.33 | Negative | 0.05 | Negative | 0.09 | Negative | No | N/A |
| 359 | N/A |  |  | 10-Aug-20 | Negative |  | 0.44 | Negative | 0.09 | Negative | 0.09 | Negative | No | N/A |
| 360 | N/A |  |  | 10-Aug-20 | Negative |  | 13.57 | Positive | 0.06 | Negative | 0.09 | Negative | No | N/A |
| 361 | N/A |  |  | 10-Aug-20 | Negative |  | 1.10 | Negative | 0.14 | Negative | 0.09 | Negative | No | N/A |
| 362 | N/A |  |  | 10-Aug-20 | Negative |  | 0.63 | Negative | 0.02 | Negative | 0.08 | Negative | No | N/A |
| 363 | N/A |  |  | 10-Aug-20 | Negative |  | 314.01 | Positive | 28.60 | Positive | 24.60 | Positive | No | N/A |
| 364 | N/A |  |  | 10-Aug-20 | Negative |  | 14.36 | Positive | 2.75 | Positive | 14.35 | Positive | No | N/A |
| 365 | N/A |  |  | 10-Aug-20 | Negative |  | 66.93 | Positive | 14.70 | Positive | 44.51 | Positive | No | N/A |
| 366 | N/A |  |  | 10-Aug-20 | Positive | 19.17 | 3.18 | Negative | 0.04 | Negative | 0.08 | Negative | No | N/A |
| 367 | N/A |  |  | 10-Aug-20 | Positive | 23.43 | 0.14 | Negative | 0.10 | Negative | 0.09 | Negative | No | N/A |
| 368 | N/A |  |  | 10-Aug-20 | Negative |  | 0.14 | Negative | 0.03 | Negative | 0.09 | Negative | No | N/A |
| 369 | N/A |  |  | 10-Aug-20 | Positive | 37.43 | 111.28 | Positive | 21.91 | Positive | 13.93 | Positive | No | N/A |
| 370 | N/A |  |  | 10-Aug-20 | Negative |  | 166.70 | Positive | 11.38 | Positive | 15.36 | Positive | No | N/A |
| 371 | N/A |  |  | 10-Aug-20 | Negative |  | 7.08 | Negative | 0.16 | Negative | 0.46 | Negative | No | N/A |
| 372 | N/A |  |  | 10-Aug-20 | Negative |  | 0.54 | Negative | 0.05 | Negative | 0.09 | Negative | No | N/A |
| 373 | N/A |  |  | 10-Aug-20 | Negative |  | 0.24 | Negative | 0.07 | Negative | 0.09 | Negative | No | N/A |
| 374 | N/A |  |  | 10-Aug-20 | Negative |  | 4.45 | Negative | 0.02 | Negative | 0.09 | Negative | No | N/A |
| 375 | N/A |  |  | 10-Aug-20 | Positive | 29.61 | 2.83 | Negative | 0.28 | Negative | 0.14 | Negative | No | N/A |
| 376 | N/A |  |  | 10-Aug-20 | Negative |  | 45.70 | Positive | 0.07 | Negative | 0.09 | Negative | No | N/A |
| 377 | N/A |  |  | 10-Aug-20 | Negative |  | 57.75 | Positive | 6.92 | Positive | 23.85 | Positive | No | N/A |
| 378 | N/A |  |  | 10-Aug-20 | Negative |  | 0.26 | Negative | 0.09 | Negative | 0.08 | Negative | No | N/A |
| 379 | N/A |  |  | 10-Aug-20 | Negative |  | 500.47 | Positive | 5.72 | Positive | 70.62 | Positive | No | N/A |
| 380 | N/A |  |  | 10-Aug-20 | Positive | 20.06 | 0.13 | Negative | 0.01 | Negative | 0.08 | Negative | No | N/A |
| 381 | N/A |  |  | 10-Aug-20 | Positive | 25.51 | 2.00 | Negative | 0.05 | Negative | 0.09 | Negative | No | N/A |
| 382 | N/A |  |  | 10-Aug-20 | Negative |  | 0.16 | Negative | 0.16 | Negative | 0.09 | Negative | No | N/A |
| 383 | N/A |  |  | 10-Aug-20 | Negative |  | 36.20 | Positive | 3.54 | Positive | 58.42 | Positive | No | N/A |
| 384 | N/A |  |  | 10-Aug-20 | Negative |  | 137.36 | Positive | 24.58 | Positive | 150.00 | Positive | No | N/A |
| 385 | N/A |  |  | 10-Aug-20 | Negative |  | 50.81 | Positive | 4.41 | Positive | 34.99 | Positive | No | N/A |
| 386 | N/A |  |  | 10-Aug-20 | Negative |  | 54.15 | Positive | 4.66 | Positive | 12.57 | Positive | No | N/A |
| 387 | N/A |  |  | 11-Aug-20 | Positive | 24.24 | 310.78 | Positive | 0.46 | Negative | 4.56 | Positive | No | N/A |
| 388 | N/A |  |  | 10-Aug-20 | Negative |  | 0.38 | Negative | 0.04 | Negative | 0.08 | Negative | No | N/A |
| 389 | N/A |  |  | 10-Aug-20 | Negative |  | 0.19 | Negative | 0.10 | Negative | 0.09 | Negative | No | N/A |
| 390 | N/A |  |  | 10-Aug-20 | Negative |  | 0.41 | Negative | 0.14 | Negative | 0.09 | Negative | No | N/A |
| 391 | N/A |  |  | 10-Aug-20 | Negative |  | 62.29 | Positive | 4.33 | Positive | 84.26 | Positive | No | N/A |
| 392 | N/A |  |  | 10-Aug-20 | Negative |  | 155.38 | Positive | 17.52 | Positive | 98.55 | Positive | No | N/A |
| 393 | N/A |  |  | 10-Aug-20 | Negative |  | 0.97 | Negative | 0.02 | Negative | 0.10 | Negative | No | N/A |
| 394 | N/A |  |  | 10-Aug-20 | Negative |  | 38.35 | Positive | 14.54 | Positive | 32.69 | Positive | No | N/A |

Ct-cycle threshold; N/A-not applicable; PCR-polymerase chain reaction

^*^Mindray CL-900i anti-SARS-CoV-2 IgG assay positive: optical-density cutoff index ≥10.0 vs. negative: cutoff index <10.0^1^.

^**^BioMérieux VidasIII assay positive: optical-density cutoff index ≥1.0 vs. negative: cutoff index <1.0^2^.

^***^Roche Elecsys Anti SARS-CoV-2 assay positive: optical-density cutoff index ≥1.0 vs. negative: cutoff index <1.0^3^.

^%^Specimen number consistent with Table 2 in main manuscript.

^#^Symptoms as reported in the medical record for this infection.

^$^Severity per WHO classification^4^. If N/A, no severity classification was conducted due to absence of serious symptoms to require hospitalization and severity assessment.

**References**

1 Mindray. *CL-900i Chemiluminescence immunoassay system.*, <<https://www.mindray.com/en/product/CL-900i.html>> (2020.).

2 bioMerieux. VIDAS SARS-CoV-2: Two immunoassay tests to detect IgM & IgG antibodies.

3 The Roche Group. *Roche’s COVID-19 antibody test receives FDA Emergency Use Authorization and is available in markets accepting the CE mark.*, <<https://www.roche.com/media/releases/med-cor-2020-05-03.htm>> (2020.).

4 World Health Organization. Clinical management of COVID-19. Available from: <https://www.who.int/publications-detail/clinical-management-of-covid-19>. Accessed on: May 31st 2020. (2020).
